# Dr. Robert Froriep

**Published:** 1861-09

**Authors:** 


DR. ROBERT FRORIEP, one of the most distinguished surgeons of Europe, formerly a professor at Jena and Berlin, lately died at Wiemar, of congestion of the brain. His surgical writings established for him, years ago, a world-wide reputation.

